# Das Hautarztverfahren nach Aufhebung des Unterlassungszwangs im Berufskrankheitenrecht

**DOI:** 10.1007/s00105-021-04776-7

**Published:** 2021-02-25

**Authors:** Peter Elsner

**Affiliations:** Klinik für Hautkrankheiten, Universitätsklinikum Jena, Erfurter Str. 35, 07743 Jena, Deutschland

**Keywords:** Prävention, Hauterkrankungen, Handekzem, Hautarztbericht, Rückfälligkeit, Prevention, Skin diseases, Hand eczema, Dermatologist’s procedure, Recurrence

## Abstract

Die Berufskrankheit (BK) Haut Nr. 5101 war bisher definiert als „schwere oder wiederholt rückfällige Hauterkrankungen, die zur Unterlassung aller Tätigkeiten gezwungen haben, die für die Entstehung, die Verschlimmerung oder das Wiederaufleben der Krankheit ursächlich waren oder sein können“. In einer weitreichenden Reform des Berufskrankheitenrechts, die zum 01.01.2021 in Kraft trat, hat der Gesetzgeber beschlossen, den „Unterlassungszwang“ als Voraussetzung für die Anerkennung einer Berufskrankheit abzuschaffen. Dieser Unterlassungszwang sollte mit dem 1972 eingeführten Hautarztverfahren und den dort etablierten Präventionsinstrumenten (dermatologische Behandlung, Sanierung des Arbeitsplatzes, Hautschutzmaßnahmen, gesundheitspädagogische Maßnahmen) verhindert werden, was in einem Großteil der Fälle auch gelang. Während die Abschaffung des Unterlassungszwangs die Anerkennung von schweren oder wiederholt rückfälligen Hauterkrankungen als Berufskrankheiten erleichtert, wird das Hautarztverfahren jedoch weiterhin Bedeutung behalten für die beruflichen Hauterkrankungen, insbesondere Handekzeme, die primär nicht die Kriterien der Schwere und/oder der wiederholten Rückfälligkeit erfüllen und die durch geeignete Behandlungs- und Präventionsmaßnahmen beherrscht werden können. Um bei einer BK-Anzeige wegen der Schwere oder wiederholten Rückfälligkeit einer Hauterkrankung Verzögerungen in der Sekundärprävention zu vermeiden, sollte gleichzeitig über einen Hautarztbericht zur BK-Anzeige der Unfallversicherungsträger die für zeitnahe Präventionsmaßnahmen und einen dermatologischen Behandlungsauftrag erforderlichen Informationen erhalten. Ein Anhaltspunkt für die Schwere könnte der Erfolg oder der Misserfolg des Hautarztverfahrens sein.

## Abstract (en)

Occupational disease (German: Berufskrankheit, BK) No. 5101 was previously defined as a “serious or recurrent skin disease that has forced a person to refrain from all activities that were or could be the cause of the development, aggravation or recurrence of the disease”. In a far-reaching reform of the occupational disease law, which came into force on 01 January 2021, the German legislature decided to abolish the “obligation to refrain” as a prerequisite for the recognition of an occupational disease. To avoid this “obligation to refrain” was the motivation to introduce the “dermatologist’s procedure” in 1972. The prevention instruments established in this procedure (dermatological treatment, workplace sanitation, skin protection measures, health education) were successful in the majority of cases. While the abolition of the “obligation to refrain” will, on the one hand, facilitate the recognition of serious or recurrent skin diseases as occupational diseases, the “dermatologist’s procedure” will, however, continue to be important for occupational skin diseases, especially cases of hand eczema, which primarily do not meet the criteria of severity and/or recurrence and which can be controlled by appropriate treatment and prevention measures. In order to avoid delays in secondary prevention when notifying an occupational disease, a supplementary dermatological report to the disease notification should provide accident insurance carriers with the information necessary for timely prevention measures and dermatological treatment. An indication for the severity of the skin disease might be the success or failure of the “dermatologist’s procedure”.

Die Berufskrankheiten der Haut führen die Berufskrankheitenstatistik in Deutschland nach wie vor an. Nach den neuesten Daten der Deutschen Gesetzlichen Unfallversicherung (DGUV) [21] erfolgten im Jahr 2019 19.883 Meldungen einer Berufskrankheit (BK) Nr. 5101, die – derzeit noch – definiert ist als „schwere oder wiederholt rückfällige Hauterkrankungen, die zur Unterlassung aller Tätigkeiten gezwungen haben, die für die Entstehung, die Verschlimmerung oder das Wiederaufleben der Krankheit ursächlich waren oder sein können“. Dass von diesen gemeldeten Erkrankungen mit 383 Fällen nur ein Bruchteil als BK anerkannt wurde, ist im Wesentlichen darauf zurückzuführen, dass im Rahmen des seit fast 50 Jahren (1972) existierenden Hautarztverfahrens (HAV) eine Vielzahl von präventiven Instrumenten eingesetzt werden kann, die dazu beitragen, den bisher rechtlich geforderten medizinisch-objektiven Unterlassungszwang als Voraussetzung für die Anerkennung einer BK 5101 zu vermeiden. Mit dem „Siebten Gesetz zur Änderung des Vierten Buches Sozialgesetzbuch und anderer Gesetze“ (7. SGB IV-ÄndG) [20] hat der deutsche Gesetzgeber kürzlich beschlossen, den Unterlassungszwang bei der BK 5101 und weiteren Berufskrankheiten abzuschaffen; begründet wurde dies damit, dass die mit dem Unterlassungszwang verfolgten Ziele, insbesondere der Ausschluss von Bagatellfällen, auf anderem Wege erreicht werden können. Diese wichtige Änderung des Berufskrankheitenrechts ist zum 01.01.2021 in Kraft getreten; damit stellt sich die Frage, wie das Hautarztverfahren unter diesen neuen Bedingungen weiterzuentwickeln ist. Im Folgenden sollen das Hautarztverfahren und die damit entwickelten Instrumente in der Sekundärprävention der BK 5101 dargestellt und deren weitere Bedeutung einerseits in der BK-Prävention, andererseits aber auch in der Minderung der BK-Folgen nach neuem Recht besprochen werden.

## Rechtsgrundlagen des § 3 der Berufskrankheiten-Verordnung und des Hautarztverfahrens (Tab. 1)

Die Gesundheitsprävention ist eine Aufgabe von höchster Priorität der UV(Unfallversicherung)-Träger; schon die 1. Berufskrankheiten-Verordnung (BKV) von 1925 enthielt eine Kannvorschrift in § 6. Wenn die Entstehung, das Wiederentstehen oder die Verschlimmerung einer gewerblichen Berufskrankheit (BK) zu befürchten war, konnte der UV-Träger eine Übergangsrente bis zur Hälfte der Vollrente so lange gewähren, wie der Versicherte die gefährdenden Tätigkeiten unterließ. Nach § 5 der 3. BKV von 1936 wurde diese Kannvorschrift in eine Sollvorschrift umgewandelt, der UV-Träger sollte bei einer drohenden BK nach Aufgabe der schädigenden Tätigkeit Übergangsrente zum Ausgleich hierdurch bedingter wirtschaftlicher Nachteile gewähren. Die Rechtsvorschrift des § 9 Absatz 6 Nr. 1 SGB (Sozialgesetzbuch) VII („Die Bundesregierung regelt durch Rechtsverordnung mit Zustimmung des Bundesrates 1. Voraussetzungen, Art und Umfang von Leistungen zur Verhütung des Entstehens, der Verschlimmerung oder des Wiederauflebens von Berufskrankheiten, …“) ist die Ermächtigungsgrundlage zum Erlass von Rechtsverordnungen zur Regelung von vorbeugenden Leistungen bei Berufskrankheiten, entspricht weitgehend der vorherigen Ermächtigung des § 551 Absatz 4 der RVO a. F. (Reichsversicherungs-Ordnung alter Fassung) und ist Ausdruck des Präventionsgedankens. Sie konkretisiert den Grundsatz des § 14 SGB VII, wonach die UV-Träger für die Prävention mit allen geeigneten Mitteln u. a. für die Verhütung von Berufskrankheiten zu sorgen haben. Diese Präventionspflicht ist seit der 7. BKV von 1968 in deren § 3 Absatz 1 Satz 1 wie folgt gefasst: „Besteht für Versicherte die Gefahr, daß eine Berufskrankheit entsteht, wiederauflebt oder sich verschlimmert, *haben* die UV-Träger dieser Gefahr mit allen geeigneten Mitteln entgegenzuwirken.“

**Table 1:** 

Datum	Rechtsakt	Präventionsvorschrift
12.05.1925	Verordnung über Ausdehnung der Unfallversicherung auf gewerbliche Berufskrankheiten	Kann-Vorschrift für Übergangsrente bei Unterlassung der gefährdenden Tätigkeit
16.12.1936	Dritte Verordnung über Ausdehnung der Unfallversicherung auf Berufskrankheiten	Soll-Vorschrift
20.06.1968	Siebente Berufskrankheitenverordnung	§ 3 (1) Besteht für Versicherte die Gefahr, daß eine Berufskrankheit entsteht, wiederauflebt oder sich verschlimmert, haben die Unfallversicherungsträger dieser Gefahr mit allen geeigneten Mitteln entgegenzuwirken. Ist die Gefahr gleichwohl nicht zu beseitigen, haben die Unfallversicherungsträger darauf hinzuwirken, daß die Versicherten die gefährdende Tätigkeit unterlassen. Den für den medizinischen Arbeitsschutz zuständigen Stellen ist Gelegenheit zur Äußerung zu geben
01.07.1972	Vertrag Ärzte-Unfallversicherungsträger	§ 41 Vorstellungspflicht beim Hautarzt: Jeder Arzt ist verpflichtet, einen Versicherten mit krankhaften Hautveränderungen, bei dem die Möglichkeit besteht, daß daraus eine Hauterkrankung durch eine berufliche Tätigkeit im Sinne der Berufskrankheitenverordnung entsteht, ..., unverzüglich einem Hautarzt vorzustellen
01.07.2013	Vertrag Ärzte-Unfallversicherungsträger	Jeder Arzt ist verpflichtet, einen Versicherten mit krankhaften Hautveränderungen, bei dem die Möglichkeit besteht, dass daraus eine Hauterkrankung durch eine berufliche Tätigkeit im Sinne der BK 5101 der Anlage 1 zur Berufskrankheitenverordnung (Schwere oder wiederholt rückfällige Hauterkrankungen, die zur Unterlassung aller Tätigkeiten gezwungen haben, die für die Entstehung, die Verschlimmerung oder das Wiederaufleben der Krankheit ursächlich waren oder sein können) entsteht, wiederauflebt oder sich verschlimmert, unverzüglich einem Hautarzt mit Formtext F 2900 – ÜV – vorzustellen
23.06.2020	Siebtes Gesetz zur Änderung des Vierten Buches Sozialgesetzbuch und anderer Gesetze	§ 9 wird wie folgt geändert: a) In Absatz 1 Satz 2 zweiter Halbsatz werden die Wörter „oder wenn sie zur Unterlassung aller Tätigkeiten geführt haben, die für die Entstehung, die Verschlimmerung oder das Wiederaufleben der Krankheit ursächlich waren oder sein können“ gestrichen

Damit verlangt der § 3 Absatz 1 ein frühes Eingreifen des UV-Trägers, um der Gefahr der Entstehung, der Verschlimmerung oder des Wiederauflebens einer BK entgegenzuwirken. Allerdings fehlte es seit 1925 an Meldekriterien des Gesetz- bzw. Verordnungsgebers für die Tatbestände des § 3, damit der UV-Träger überhaupt erst von einer „drohenden BK“ erfuhr und tätig werden konnte. Unter Beachtung dieser rechtlichen Voraussetzungen war es möglich, das Hautarztverfahren (HAV) als ein vertraglich zwischen Unfallversicherungen und Ärzteschaft vereinbartes Verfahren einzuführen. Dies geschah zum 01.07.1972 in dem „Abkommen Ärzte-Berufsgenossenschaften“ (heute: „Vertrag Ärzte-Unfallversicherungsträger“). Insbesondere Siegfried Borelli hat sich um das HAV maßgebliche Verdienste erworben und Tausende von Patienten mit Berufsdermatosen untersucht; bereits 1971 forderte er aus Sicht der Dermatologie die Schaffung von „Haut-Durchgangsärzten“ bei Hautberufserkrankten [2]. Während das HAV seinerzeit für alle Hautkrankheiten der damals geltenden 7. BKV vom 20.06.1968 galt, wurde es 2013 auf die BK 5101 eingeschränkt, nachdem angeregt worden war, das HAV auch für die Prävention der seinerzeit zur Einführung anstehenden BK 5103 zu nutzen [9].

## Präventive Maßnahmen im Kontext des Hautarztverfahrens (Tab. 2)

Mit der Einführung des Hautarztverfahrens 1972 wurde die Vorstellung beim Hautarzt und dessen Berichterstattung an den Unfallversicherungsträger eine verbindliche Aufgabe der Ärzteschaft. Die Umsetzung der durch den Hautarztbericht gewonnenen Informationen in ein koordiniertes Präventionshandeln war jedoch anfangs je nach Unfallversicherungsträger unterschiedlich. Durch eine von J. Schwanitz, Osnabrück, in Zusammenarbeit mit der Berufsgenossenschaft für Gesundheitsdienst und Wohlfahrtspflege (BGW) in den 1990er-Jahren durchgeführte Studie im seinerzeit von Berufsekzemen besonders betroffenen Friseurgewerbe [12], die später durch Untersuchungen zur Altenpflege [13] ergänzt wurde, konnten beispielhaft Konzepte für die sekundäre Individualprävention entwickelt werden, die später von anderen Unfallversicherungsträgern in Anpassung an ihre spezifischen Bedürfnisse übernommen wurden [1]. Im Jahr 1999 wurde eine Optimierung des HAV durch den seinerzeitigen Hauptverband der Gesetzlichen Berufsgenossenschaften (HVBG) initiiert und in Zusammenarbeit mit der Dermatologie erfolgreich unter Berücksichtigung von Erkenntnissen der Präventionsforschung aktualisiert [6]. Das 1972 eingeführte Früherkennungsverfahren entwickelte sich so zum Frühinterventionsverfahren mit effizienten Präventions- und Therapieverfahren [10]. Neben technischen und organisatorischen Interventionen durch Präventionsdienste der UV-Träger am Arbeitsplatz, ambulanten gesundheitspädagogischen Interventionsmöglichkeiten wie der Beratung in spezialisierten Zentren der Berufsgenossenschaften [11] und Hautschutzzentren [8, 15] und der kurzfristigen Ausstattung mit evidenzbasiert ausgewählten Hautmitteln wurde auf der Basis einer von der DGUV unterstützten Multicenterstudie ab 2005 deutschlandweit ein stationäres tertiäres Individualpräventionsprogramm etabliert [14, 18], das auch über längere Nachbeobachtungszeiten einem Großteil der Patienten die Weiterarbeit im Beruf ermöglichte [4]. Daneben entwickelte sich das Hautarztverfahren auch zu einem wichtigen Instrument in der Begutachtung der BK 5101. Der Gutachter hatte zu prüfen, ob medizinisch-objektiv ein Unterlassungszwang für die gefährdende Tätigkeit bestand, d. h. ob die Gefahr durch die Maßnahmen des Hautarztverfahrens nicht beseitigt werden konnte [7]. Erst wenn diese Maßnahmen auch tatsächlich durchgeführt worden und nachweislich fehlgeschlagen waren, konnte der Unterlassungszwang als Kriterium für das Vorliegen der BK 5101 neben der Schwere und der wiederholten Rückfälligkeit bestätigt werden. Die im Rahmen des Hautarztverfahrens erfolgenden dermatologisch dokumentierten Arbeitsversuche boten so auch die Möglichkeit zu einer verbesserten Beurteilung der beruflichen Kausalität einer Hauterkrankung.

**Table 2:** 

Instrument	Zweck(e)	Umsetzung
Berichterstattung an den UV-Träger	Frühzeitige Erfassung der konkreten Gefahr einer BK	Hautarztbericht/Stellungnahme zur Prävention [6]
Dermatologische Heilbehandlung	Frühzeitige leitliniengerechte dermatologische Behandlung „mit allen geeigneten Mitteln“	Behandlungsauftrag des UV-Trägers als Teil des „Verfahrens Haut“ [17]
Berichterstattung an den UV-Träger	Monitoring des Behandlungserfolgs	Hautarzt-Verlaufsbericht [6]
Hautschutz- und Handschuhberatung	Optimierung des beruflichen Hautschutzes; frühzeitige Umsetzung am Arbeitsplatz durch Erstausstattung	Schulungs- und Beratungszentren der UV-Träger [11], Hautschutzzentren [14, 15]
Identifizierung von Hautschädigungen am Arbeitsplatz	Nutzung möglicher technischer und organisatorischer Maßnahmen zur Reduzierung der Hautbelastung am Arbeitsplatz (Elimination/Substitution)	Ermittlungen des Präventionsdienstes des UV-Trägers am Arbeitsplatz [3]
Gesundheitspädagogische Intervention	Veränderungen des individuellen Hautschutz- und Hautpflegeverhaltens	Ambulante sekundäre Individualpräventionsseminare (SIP) [19]
Multidisziplinäre dermatologisch/gesundheitspädagogische stationär/ambulante Intervention	Regeneration des Barriereschadens; erweiterte Diagnostik und stadiengerechte Therapie; gesundheitspädagogische und -psychologische Interventionen; Umsetzung von individuellen Hautschutzmaßnahmen unter ergotherapeutischer Begleitung	Stationäres Heilverfahren (tertiäre Individualprävention) [18]

*UV-Träger* Unfallversicherungsträger, *BK* Berufskrankheit

## Weiterentwicklung des Hautarztverfahrens nach Wegfall des Unterlassungszwangs im Berufskrankheitenrecht

In der Vergangenheit war es eher selten, dass Patienten mit schweren und/oder wiederholt rückfälligen Hauterkrankungen beim Hautarzt vorgestellt wurden und bereits zum Zeitpunkt der Erstvorstellung der Unterlassungszwang bejaht wurde, etwa weil ein nicht meidbares starkes Allergen am Arbeitsplatz vorkam. Dies war etwa der Fall, wenn eine schwere generalisierte Urtikaria mit Anaphylaxie bei Alleinköchen durch ein nicht meidbares Nahrungsmittelallergen auftrat [5]. In diesen Fällen war – und ist auch in Zukunft – eine Berufskrankheiten-Anzeige nach § 202 SGB VII an den UV-Träger verpflichtend; anders als durch Unterlassung der Tätigkeit war die Gefahr für den Versicherten nicht zu beseitigen.

Mit dem Wegfall des Unterlassungszwangs werden auch schon zum Zeitpunkt der Erstvorstellung beim Hautarzt schwere und/oder wiederholt rückfällige Hautkrankheiten wegen des begründeten Verdachts einer BK 5101 anzeigepflichtig, selbst wenn die Tätigkeit unter geeigneten Präventionsmaßnahmen fortgesetzt werden kann. Zu der Beurteilung der Schwere sind die vom Verordnungsgeber im Merkblatt zur BK 5101 genannten Kriterien heranzuziehen, nämlich die klinische Symptomatik nach Morphe und Beschwerdebild, Ausdehnung, Verlauf und Dauer der Erkrankung und aufgrund der Ausprägung der beruflich verursachten Allergien [22]. Weitergehende Konkretisierungen zum Kriterium der Schwere werden derzeit von der Arbeitsgruppe „Bamberger Empfehlung“ der Arbeitsgemeinschaft für Berufs- und Umweltdermatologie (ABD) in Kooperation mit der DGUV erarbeitet [7]. Für die so gemeldeten Fälle wird der UV-Träger ein Berufskrankheiten-Feststellungsverfahren einleiten müssen, das aufgrund des erheblichen Ermittlungsaufwandes und der Einholung einer berufsdermatologischen Begutachtung erfahrungsgemäß erhebliche Zeit in Anspruch nehmen kann. Damit in der Zwischenzeit die dermatologische Behandlung bereits nach § 3 BKV „mit allen geeigneten Mitteln“ eingeleitet werden kann, sollte der Hautarzt gleichzeitig einen Heilbehandlungsantrag an den Unfallversicherungsträger stellen. Dies wird ermöglicht durch eine kurzfristige Änderung des Vertrages Ärzte-Unfallversicherungsträger; danach soll in § 41 Abs. 2 folgender Satz eingefügt werden: „Der Hautarztbericht F 6050 ist auch zu erstatten, wenn zum Zeitpunkt der Untersuchung bereits der begründete Verdacht auf das Vorliegen einer Berufskrankheit im Sinne der BK-Nr. 5101 besteht.“ Nach § 45 Vertrag Ärzte-Unfallversicherungsträger teilt der Unfallversicherungsträger dem anzeigenden Arzt unverzüglich mit, ob und ab welchem Zeitpunkt Heilbehandlung zulasten des Unfallversicherungsträgers durchzuführen ist. Da der Hautarzt danach auch bei einer BK-Anzeige zum Hautarztbericht verpflichtet bleibt, dürfte er anders als im BK-Feststellungsverfahren weiterhin berechtigt sein, Tests durchzuführen, die zur Klärung des Ursachenzusammenhangs zwischen der Hauterkrankung und der beruflichen Tätigkeit erforderlich sind; dies ist im BK-Feststellungsverfahren üblicherweise dem vom UV-Träger beauftragten Gutachter vorbehalten. Im Einzelfall sollte ggf. die Genehmigung für aus Sicht des behandelnden Hautarztes erforderliche Testungen zulasten des Unfallversicherungsträgers eingeholt werden. Mit einer bloßen BK-Anzeige nach § 202 SGB VII erhält der Unfallversicherungsträger nicht die ausführlichen, für eine rasche Prävention noch vor Abschluss des BK-Feststellungsverfahrens wichtigen Informationen aus dem optimierten Hautarztbericht. Es ist daher erfreulich, dass mit der geplanten Änderung in § 41 Abs. 2 des Vertrages Ärzte-Unfallversicherungsträger sowohl BK-Anzeige als auch Hautarztbericht parallel gehen können.

Die meisten Berufsdermatosen betreffen jedoch zumindest anfangs durchaus gut therapierbare, noch nicht die Kriterien der Schwere erfüllende Hauterkrankungen, insbesondere Handekzeme; für sie bleibt die Verpflichtung zur Erstellung des Hautarztberichtes nach § 41 Vertrag Ärzte-Unfallversicherungsträger unverändert bestehen, und die Präventionsmaßnahmen im Rahmen des Hautarztverfahrens (Tab. 2) können vom Unfallversicherungsträger in bewährter Weise eingeleitet werden. Begrenzt wird das Hautarztverfahren ab dem 01.01.2021 allerdings durch den Umstand, dass auch eine klinisch leichte Hauterkrankung allein wegen ihrer Dauer als schwer einzustufen sein kann, wenn eine ununterbrochene Behandlungsbedürftigkeit von 6 und mehr Monaten gegeben ist [22]. In diesem Fall wird der Unfallversicherungsträger bei im Rahmen des Hautarztverfahrens behandelten Versicherten ein BK-Feststellungsverfahren einleiten müssen. Auch in diesen Fällen wird vom Hautarzt darauf zu achten sein, dass die im Hautarztverfahren eingeleitete Behandlung möglichst ununterbrochen weitergeführt werden kann.

Im Falle der Anerkennung einer BK 5101 als Ergebnis des Feststellungsverfahrens bleibt der Unfallversicherungsträger – anders als im Hautarztverfahren als sog. „kleinem Versicherungsfall“ – auf Dauer für die Behandlung der beruflich verursachten oder verschlimmerten Hauterkrankung zuständig, wobei die Abgrenzung zwischen endogenen Krankheitsverläufen und beruflich exogenem Schaden wie bisher schon herausfordernd sein kann [16]. Bei hautgefährdend weiterarbeitenden Versicherten kommt als präventiver Aspekt für die Unfallversicherung die Verpflichtung aus § 3 BKV zum Tragen, die Verschlimmerung einer anerkannten BK zu verhüten. Hier können – dann zeitlich anders als im Hautarztverfahren unbeschränkt – dieselben „geeigneten Mittel“ zum Einsatz kommen wie im Hautarztverfahren, d. h. ambulante und stationäre dermatologische Behandlung, präventive Maßnahmen am Arbeitsplatz, Ausstattung mit Hautmitteln, ambulante und stationäre gesundheitspädagogische Schulungen zur Verhaltensprävention. Mit der neuen gesetzlichen Regelung des § 9 Abs. 8 SGB VII werden die Versicherten verpflichtet, an diesen Maßnahmen mitzuwirken.

## Schlussfolgerung

Das Hautarztverfahren ist ein seit 1972 bewährtes Präventionsverfahren bei der BK 5101 und das bisher einzige „Vorfeldverfahren“ bei einer Berufskrankheit. Mit der Abschaffung des „Unterlassungszwangs“ im Berufskrankheitenrecht zum 01.01.2021 werden berufliche Hautkrankheiten, die schwer oder wiederholt rückfällig sind, unverzüglich mittels einer Berufskrankheitenanzeige gemeldet werden müssen, auch wenn diese nicht zur Aufgabe der beruflichen Tätigkeit zwingen und die Tätigkeit fortgeführt wird. Das Hautarztverfahren wird jedoch dadurch keineswegs überflüssig, denn das dermatologische Berufskrankheitengeschehen wird weiterhin geprägt sein von vielen beruflichen Hauterkrankungen, insbesondere Handekzemen, die primär nicht die Kriterien der Schwere und/oder der wiederholten Rückfälligkeit erfüllen und durch geeignete Behandlungs- und Präventionsmaßnahmen beherrscht werden können. Für die Fälle, bei denen früher als in der Vergangenheit eine verpflichtende Berufskrankheiten-Anzeige erfolgen und ein Berufskrankheiten-Feststellungsverfahren eingeleitet werden muss, sollten allerdings pragmatische Regelungen gefunden werden, damit über einen ergänzenden hautärztlichen Bericht zur BK-Anzeige die Unfallversicherungsträger bereits zeitnah sekundäre Präventionsmaßnahmen einleiten und lückenlos einen Behandlungsauftrag an den Dermatologen erteilen können.

## Fazit für die Praxis

- Mit Inkrafttreten des neuen Berufskrankheiten(BK)-Rechts sind beruflich verursachte schwere und/oder wiederholt rückfällige Hauterkrankungen bei begründetem Verdacht als BK 5101 an den Unfallversicherungsträger zu melden, ohne dass ein Unterlassungszwang vorliegen muss.
- Ein Hautarztbericht ist weiterhin zu erstellen für berufliche Hauterkrankungen, bei denen die Möglichkeit der Entstehung einer BK 5101 besteht, also für Hauterkrankungen, die noch nicht schwer oder wiederholt rückfällig sind.
- Die im Hautarztverfahren erfolgreich etablierten Präventionsinstrumente (dermatologische Behandlung, technische und organisatorische Maßnahmen am Arbeitsplatz, Hautschutzmaßnahmen, gesundheitspädagogische Maßnahmen) bleiben für die Minderung der BK-Folgen wichtig; neuerdings sind die Versicherten gesetzlich verpflichtet, daran mitzuwirken.
- Über einen neu einzuführenden ergänzenden hautärztlichen Bericht zur BK-Anzeige sollten die Unfallversicherungsträger die erforderlichen Informationen erhalten, um bereits während des BK-Feststellungsverfahrens zeitnah sekundäre Präventionsmaßnahmen einleiten und einen Behandlungsauftrag an den Dermatologen erteilen zu können.
